# No Risk, No Differences. Neural Correlates of Temperamental Traits Revealed Using Naturalistic fMRI Method

**DOI:** 10.3389/fpsyg.2019.01757

**Published:** 2019-08-06

**Authors:** Maria Bierzynska, Pamela Anna Sobczak, Anna Kozak, Maksymilian Bielecki, Jan Strelau, Malgorzata Maria Kossut

**Affiliations:** ^1^Laboratory of Neuroplasticity, Nencki Institute of Experimental Biology, Warsaw, Poland; ^2^Faculty of Psychology, SWPS University of Social Sciences and Humanities, Warsaw, Poland

**Keywords:** temperament, Regulative Theory of Temperament, fMRI, dynamic stimuli, limbic system

## Abstract

The main goal of this study was to identify the moderating role of temperamental traits, as defined by Strelau’s Regulative Theory of Temperament (RTT), in explaining brain activity evoked by video stimuli of varying stimulatory value. fMRI scans were performed in a group of 61 young females in the luteal phase of the menstrual cycle. The validity of stimulus selection had been verified prior to the main study by collecting declarative measures of affective reactions, including valence, arousal, and basic emotions ratings. The choice of dynamic and complex video-stimuli allowed us to induce high levels of arousal effectively. Three categories of movies used in the experiment included neutral, low arousing, and highly arousing scenes. Movies classified into the last category depicted extreme-sport activities allowing us to confront the subjects with recordings potentially life-threatening situations. Results of the study revealed that activation of orbitofrontal cortex in highly arousing conditions is linked to the levels of activity, while traits of perseverance and emotional reactivity were negatively correlated with the BOLD signal in this structure. Low arousing movies evoked higher activation of the amygdala and left hippocampus in emotionally reactive subjects. Obtained results might be coherently interpreted in the light of RTT theory, therefore providing its first validation using functional brain imaging.

## Introduction

From a historical perspective, temperament is one of the earliest theoretical constructs investigated by psychologists. Despite decades of theoretical and empirical developments, its core definition remained unchanged. Temperament is understood as a set of basic, biologically determined personality traits. Most influential theories of temperament include: Buss and Plomin Behavior Genetics-Oriented Model of Temperament (Buss and Plomin, 1975, 1984), The Goldsmith Approach (Goldsmith and Campos, 1982; Goldsmith et al., 1987), Behavioral Inhibition (Kagan et al., 1988), The Rothbart Approach (Rothbart and Derryberry, 1981), Thomas and Chess Nine Temperament Characteristics (Thomas and Chess, 1977). Temperamental traits might also be incorporated as parts of broader personality models as it is the case in PEN (Eysenck, 1947, 1973) or Big Five (Costa and McCrae, 1980).

In the current study, we decided to use the Regulatory Theory of Temperament (RTT) proposed by Jan Strelau. According to this theory, temperament is a set of basic, primarily biologically determined and relatively stable personality traits, which apply to the formal characteristics of behavior. There are two types of temperamental characteristics described in RTT: energetic and temporal. Briskness [the tendency to react quickly, to keep up a high tempo of performing activities, and to shift easily in response to changes in the surroundings from one behavior (reaction) to another] and perseverance [the tendency to continue and to repeat behavior after cessation of stimuli (situations) evoking this behavior] are classified as energetic traits. Sensory sensitivity (the ability to react to sensory stimuli of low stimulative value), endurance (the ability to react adequately in situations demanding long-lasting or high stimulative activity and under intensive external stimulation) and activity (the tendency to undertake behavior of high stimulative value or to supply by means of behavior strong stimulation from the surroundings) are considered temporal traits (Strelau, 1982; Strelau et al., 2002).

Contrary to other theories of temperament (for example PEN), Strelau did not hypothesize about any specific neurophysiological mechanism responsible for traits manifestation. Nevertheless, he stated that behavioral activation plays a major role in reactions related to temperamental traits. As a consequence, the functional significance of temperament can be most clearly demonstrated when individuals are confronted with stressful situations or excessive environmental demands (Strelau, 1985). Furthermore, the author of RTT postulated that the level of activation is regulated by biochemical and physiological processes (Strelau, 1985) which determine each person’s “neurochemical identity.”

Thus, even though the biological processes underlying individual differences in temperament are postulated within the RTT, particular traits are not ascribed to specific mechanisms (Strelau and Zawadzki, 1993; Strelau, 1998). Furthermore, no neuroimaging studies were ever performed, and only a few studies have investigated this problem on behavioral genetics level (Dragan and Oniszczenko, 2005, 2006; Oniszczenko and Dragan, 2012).

One of these studies (Zawadzki and Strelau, 1997; Strelau et al., 2002), aimed at assessing genetic and environmental contributions to the temperament traits, measured using three different questionnaires: the Formal Characteristics of Behavior – Temperament Inventory (FCB-TI) (perseverance, emotional reactivity; Zawadzki and Strelau, 1995), Windle and Lerner (1986) DOTS-R (approach-withdrawal, mood quality), and Buss and Plomin (1984) EAS-TS (distress, fear, and anger). The analysis was performed using data from a joint Polish and German sample of 1555 mono- and dizygotic twins. The self-report data indicated that an additive genetic factor explained about 40% of the phenotypic variance in traits measured by RTT. The differences among the remaining inventories were small, but, estimates of genetic influence were the highest for the FCB-TI scales in self- and peer reports. This result convincingly supports both the theoretical validity of RTT and Strelau’s claims about the strong biological underpinnings of temperamental traits defined by his theory.

Despite the crucial role of biological bases postulated in all theories of temperament, neuroimaging studies are still struggling to identify neural processes underlying temperamental traits. The literature of fMRI studies focused on temperamental traits is broad, however, obtained results are often contradictory (Yarkoni, 2015). Task-based fMRI studies rest on the assumption that differences in temperamental traits may affect task processing (Yarkoni, 2015). One of the earliest studies using affective stimuli was performed by Canli et al. (2001), who used pictures in order to examine the differences in brain activation depending on the valence of the presented material. His results showed that neuroticism is linked to the increased levels of activation in middle frontal and temporal gyri while watching negative pictures, while extraversion predicts higher activation in the middle frontal gyri, cingulate and middle temporal gyri, amygdala, and the basal ganglia during the presentation of positive stimuli.

Canli discoveries concerning affect-processing regions were further corroborated in other studies using static affective pictures (e.g., Kehoe et al., 2011) and emotional expressions (e.g., Canli et al., 2002). Kennis et al. (2013) in their review, suggested that more sophisticated paradigms are required in neuroimaging studies of individual differences in temperament, including procedures relying on the presentation of dynamic stimuli. Movies are known to evoke stronger physiological arousal than photographs (Gross and Levenson, 1995; Sato and Yoshikawa, 2007). Due to their dynamic nature, they convey more information which enables a participant to understand a presented situation from a broader perspective, situating themself in a psychological state close to “real-life situation” (Rottenberg et al., 2007; Schaefer et al., 2010; Shimamura et al., 2013; Goldberg et al., 2014), which leads to a stronger emotional response (Westermann et al., 1996; Hasson et al., 2010).

All the results presented above determined our decision to use movie clips as the mean of eliciting emotional states in our subjects. Based on the premises of RTT, we assumed that finding neural correlates of temperamental traits should include conditions evoking the highest possible arousal levels. To achieve that goal we decided to present videos of people involved in extreme sport (and potentially even life-threatening) activities. RTT states explicitly that temperament plays a regulative role in providing stimulation which suits individual needs (Strelau, 2009). One of the ways in which an individual can obtain the most appropriate amount of stimulation is the decision to choose certain sport discipline. The connection between features of temperament defined by RTT and engaging in extreme sports is well established. The trait that is proved to play a major role in the sports discipline selection is emotional reactivity. Research showed that low reactivity facilitates better adaptation in extreme situations (Eliasz, 1981; Klonowicz, 1984; Gracz and Sankowski, 2000; Studenski, 2004). Studies conducted on glider pilots showed that this group, in comparison to the general population, is characterized, besides low emotional reactivity, by high activity and briskness (Glenc, 2006). Moreover, the connection between temperamental traits and sports preferences becomes more pronounced as the level of physical danger related to certain activity grows (Zawadzki and Strelau, 1997; Studenski, 2004; Glenc, 2006; Terelak and Jońca, 2008).

We used an experimental design in which movies differing in their arousing properties (extreme vs. low arousing sports vs. control movies) were shown to subjects in order to demonstrate the role of temperament in explaining patterns of brain activity. Based on the assumptions of RTT, we expected extreme sports to be appetitive stimuli for subjects with low emotional reactivity and high activity. In particular, we expected these traits to be correlated with increased activity of the reward system structures: nucleus accumbens, amygdala, and orbitofrontal cortex. On the other hand, the opposite pattern should be detectable in subjects with high emotional reactivity, as for this group, high levels of stimulation are aversive.

## Materials and Methods

### Subjects

Sixty-one right-handed female subjects (mean age: 23 ± 2.6) with no history of neurological or psychiatric disorders, participated in the study. Given the planned sample size, we decided to recruit participants of one sex only to avoid potential confounding effects that could not be effectively controlled. The decision to invite females was motivated by the fact that female samples are usually characterized by a larger proportion of high-reactiveness scores (Strelau and Zawadzki, 1993) and our goal was to maximize the variability of temperamental traits relevant for stimulation processing.

Most of the subjects were college students. All participants gave their informed consent prior to the start of the experiment. A local research ethics committee at the SWPS University of Social Sciences and Humanities approved the experimental protocol of the study. Subjects received financial gratification in the amount of PLN 100 (approximately EUR 24).

In addition, all participants were set up to enter the experiment in the MRI scanner during a specific phase of the menstrual cycle, which was the luteal phase, to prevent changes in the pattern of brain activity related to the differences in sex-hormones’ levels (Dreher et al., 2006; Frank et al., 2010; Gingnell et al., 2012).

### Formal Characteristics of Behavior – Temperament Inventory

Formal Characteristics of Behavior – Temperament Inventory (Zawadzki and Strelau, 1997) is a self-report questionnaire measuring all dimensions of individual differences defined by Strelau’s RTT. It consists of 120 yes-no statements grouped into six scales: Briskness (Cronbach’s α = 0.77), Perseverance (Cronbach’s α = 0.79), Sensory Sensitivity (Cronbach’s α = 0.73), Emotional Reactivity (Cronbach’s α = 0.83), Endurance (Cronbach’s α = 0.85), and Activity (Cronbach’s α = 0.83).

Formal Characteristics of Behavior – Temperament Inventory was administered to the subjects before the fMRI procedure. The final sample of participants was selected from a larger pool of students to maximize the variance of the emotional reactivity and activity scores. The scores on all scales of FCB-TI were approximately normally distributed and followed the expected pattern of inter-correlations reported for the normative sample in the questionnaire handbook. Thanks to the demographic homogeneity of the study group, there was no need to normalize or transform obtained results; therefore, in all subsequent analyses, raw scores were used.

### Behavioral Procedure

The behavioral data acquisition was performed using a dedicated online platform developed by the Laboratory of Brain Imaging at Nencki Institute^1^.

The behavioral procedure consisted of a presentation and rating of 18 short movies used in the fMRI experimental procedure (details are provided in section “fMRI Experimental Procedure”). Movies were presented in pseudorandomized order. After each of them, the participant had to answer questions assessing the intensity of basic emotions (happiness, anger, sadness, fear, disgust), valence and arousal evoked by the stimulus (each measured using a 7-point scale). The exact duration of the procedure depended on the speed of participants’ reactions as the task was self-paced.

Participants were asked to complete this part of the procedure at home (it was available online) not later than 1 week after the scanning session. To standardize the conditions, we instructed participants to provide all the ratings within one session and using a desktop computer or a laptop (not a mobile device). Valid results were obtained from 43 subjects (70.5% of the total sample).

Behavioral results were analyzed using Microsoft Excel and JAMOVI statistical package^2^. Ratings of individual movies were averaged for each subject and stimulus category (high vs. low arousal vs. control movies) and submitted to univariate repeated measures ANOVA. This procedure was repeated for each of the seven rating scales. *Post hoc* tests with Bonferroni correction were used as follow-up analysis.

### fMRI Experimental Procedure

**FIGURE 1:**
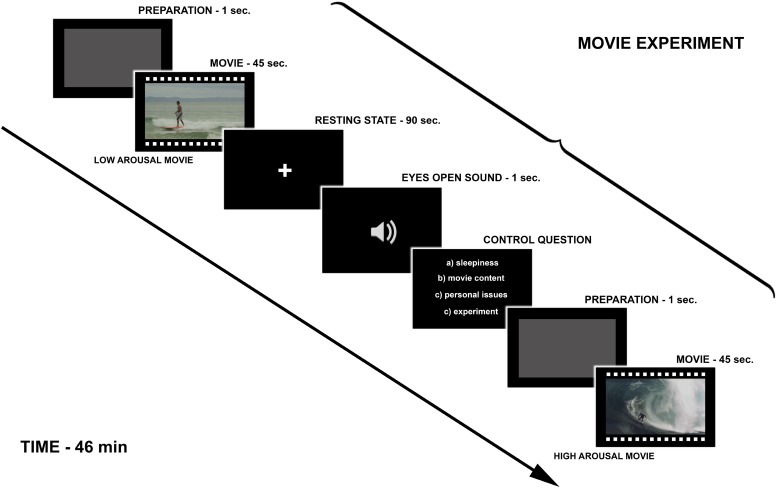
Schema of the protocol including: experimental design and single trial timings.

**FIGURE 2:**
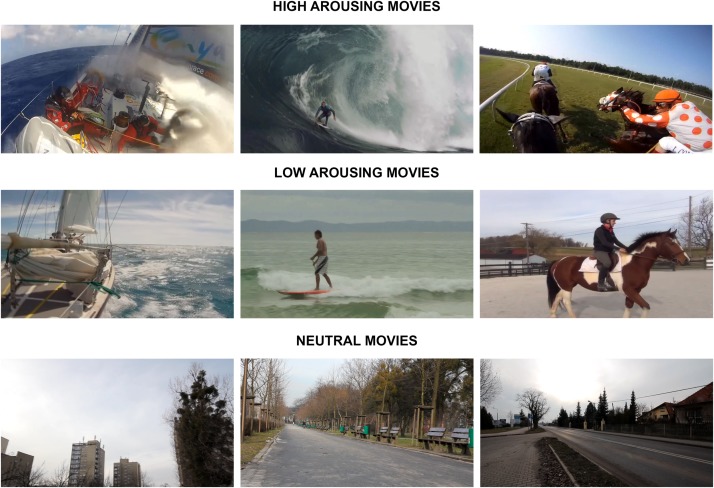
Exemplary images taken from each category of movies used in the current study. The high and the low arousing movies were selected from already existing movies, which were available on YouTube on Creative Commons license. The neutral movies were created by the authors of the study.

The experimental design (Figure 1) was based on Eryilmaz et al. (2011) study in which the impact of transient emotions on functional connectivity was examined. During the fMRI experiment, participants watched 18 movies from three categories (Figure 2). The high and the low arousing movies were selected from already existing movies, which were available on YouTube on Creative Commons license. The neutral movies were created by the authors of the study. We used six movies in each category. Eighteen trials were presented in pseudo-random order. The whole fMRI procedure lasted 46 min split into two parts with a short break in between. During the break the experimenter made sure if the participant felt well and if there was any contraindication to continue the experiment.

Each trial began with an empty gray screen (duration 1 s), which was a sign to prepare for watching the stimulus (movie). Then the 45-s movie was presented and after each movie participants were asked to close their eyes for 90 s when the measurement of the resting state activity was obtained (these results are not reported in this article). At the end of the resting state period, participants heard a sound signal, which prompted them to open their eyes and answer a control question (5 s), which came up on the screen (the control question was about the nature of subjects’ thoughts during the resting state: thoughts about sleepiness, movie content, personal issues, experiment environment). The answers were given with the right hand through the ResponsePad (NNL Response Grip)^3^. The aim of the control question was to ensure that the subject did not fall asleep. Figure 1 presents the Schema of the fMRI experiment.

Every participant underwent a training procedure before the main experiment. The training allowed participants to get accustomed to the response pad used for the control questions and to learn to react to the sound signal indicating the end of the resting-state recording.

### Image Acquisition and Data Analysis

Whole brain imaging was performed with a 3-Tesla MRI scanner (Siemens Magnetom Trio TIM, Erlangen, Germany) equipped with 32-channel phased array head coil. Head movements were minimized with cushions placed around the participants’ heads.

Functional data were acquired using a T2^*^-weighted gradient echo planar imaging (EPI) sequence with the following parameters: time repetition = 1.100 ms; time echo = 28 ms; flip angle = 80°; matrix size = 96 × 96; field of view = 190 mm; in-plane resolution: 1.98 mm × 1.98 mm; and 48 axial slices, with 3 mm slice thickness with no gap between slices. Detailed anatomical data of the brain were acquired with T1-weighted (time repetition = 2530 ms; time echo = 3.32 ms) sequences with isotropic voxel size (1 mm × 1 mm × 1 mm). For each subject, the functional run consisted of 1226 volumes lasting 46 min (split into two parts).

Statistical Parametric Mapping (SPM8, Wellcome Trust Center for Neuroimaging, London, United Kingdom) running on MATLAB 2012 (The Mathworks Inc., Natick, MA, United States) was used for data processing and statistical analyses. First, functional images were motion corrected. Then, structural images from single subjects were coregistered to the mean functional image. High-dimensional Diffeomorphic Anatomical Registration through Exponentiated Lie Algebra (DARTEL) was used to create a group-specific template and flow fields based on segmented tissue from T1w images. The functional images were normalized using compositions of flow fields and group-specific template to a 2 mm isotropic voxel size. Finally, the normalized functional images were smoothed with a 5 mm isotropic Gaussian kernel. In the first-level statistical analysis, experimental runs for each subject were merged into a single model. All the experimental events were modeled and head movement parameters were entered as covariates into the design matrix. Movies, resting state and control question were modeled. All stimulus functions were convolved with the canonical hemodynamic response basis function (HRF). On the second-level analysis, *t*-tests depicting the main effect of movies, rest and movies categories were computed for the whole group.

All the reported data were a family wise error (FWE) corrected for multiple comparisons at the voxel level and a significance threshold of *p* < 0.05 was applied. For each cluster, the main peak of activation with a corresponding *t*- and *p*-values, as well as the brain structure name, are provided in the tables.

The second level regression concerning the effect of temperamental traits in whole-brain analysis did not reveal any significant results. Therefore, the relationship between the temperamental traits and brain activity during presentations of each movie category was further studied using the region of interest (ROI) analysis. ROIs were defined using Automated Anatomical Labeling atlas (AAL)^4^. Due to the sparsity of data on correlates of temperamental differences our choice of studied structures was determined by existing literature on emotional processing and included: frontal and prefrontal areas (Canli et al., 2001; Miller and Cohen, 2001; Cremers et al., 2009), insula (Paulus et al., 2003), precuneus and lingual gyrus (Adelstein et al., 2011), structures within the limbic system, and the basal ganglia (Canli et al., 2001). In the second step β values from predefined structures was extracted using MarsBaR software (Brett et al., 2002) for every condition of movie watching (neutral movies, low arousing, and high arousing movies) and correlated with the raw scores from FCB-TI.

## Results

### Behavioural Results

Analysis of behavioral results confirmed the existence of the clear differences among the three categories of movies used in the study. A significant effect of movie-category was found for each dimension used in the rating procedure. Observed mean scores followed the expected pattern, in particular, extreme-sport movies evoked highest levels of arousal and fear. All the descriptive statistics, as well as details of ANOVA results, are available in Table 1.

**TABLE 1:** Descriptive statistics and results of the repeated measures ANOVA comparing ratings of the three categories of stimuli.

		**Neutral movies**		**Low arousal movies**		**High arousal movies**		**Repeated measures ANOVA**		
**Rating scale**	**Range of scores**	**M**	**SD**	**M**	**SD**	**M**	**SD**	***F*(2,84) =**	***p***	**h^2^_*p*_**
Happiness	1–7	1.37	0.48	2.91	1.05	3.41	1.50	59.50	<0.001	0.59
Anger	1–7	1.39	0.72	1.15^a^	0.35	1.12^a^	0.36	6.45	0.002	0.13
Sadness	1–7	1.41	0.60	1.16^a^	0.37	1.06^a^	0.14	9.27	<0.001	0.18
Fear	1–7	1.13^a^	0.27	1.37^a^	0.51	2.81	1.32	76.70	<0.001	0.65
Disgust	1–7	1.23	0.42	1.02^a^	0.09	1.06^a^	0.21	7.79	<0.001	0.16
Valence	−3 to +3	−0.23	0.73	0.91	0.66	1.33	0.99	49.90	<0.001	0.54
Arousal	1–7	1.45	0.60	2.46	0.82	4.00	1.26	135.70	<0.001	0.76

*All post hoc comparisons (with Bonferroni correction) are significant at p < 0.05 level, unless equality of means between movie categories is indicated by the shared letter in the superscript next to the mean score.*

### Functional Neuroimaging Results

The main purpose of the study was to use subjects’ individual scores in order to explore the role of temperament in explaining brain activation evoked by different categories of video-stimuli. However, for the sake of completeness, we start our analysis with examining the basic effects of resting and movie-watching as well as the differential effects of movie categories – regardless of the temperamental traits.

#### Main Effects of Movies and Rest

**FIGURE 3:**
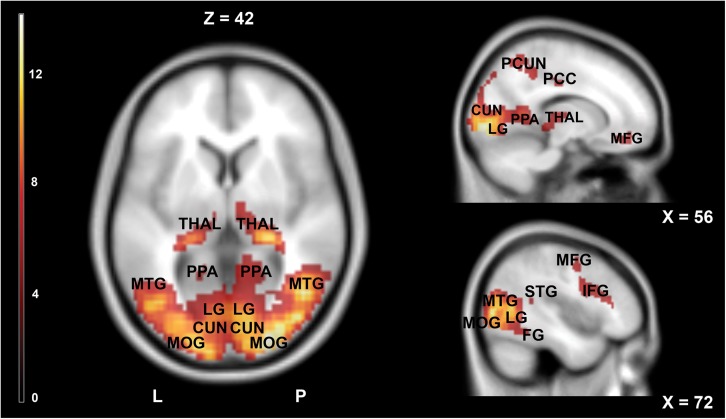
Brain activity pattern in the whole group (*n* = 61) associated with the main effect of movies (movies vs. rest). CUN, cuneus; IFG, inferior frontal gyrus; LG, lingual gyrus; MFG, middle frontal gyrus; MOG, middle occipital gyrus; MTG, middle temporal gyrus; PCC, posterior cingulate cortex; PCUN, precuneus; PPA, parahippocampal gyrus; THAL, thalamus; L, left side; R, right side.

**FIGURE 4:**
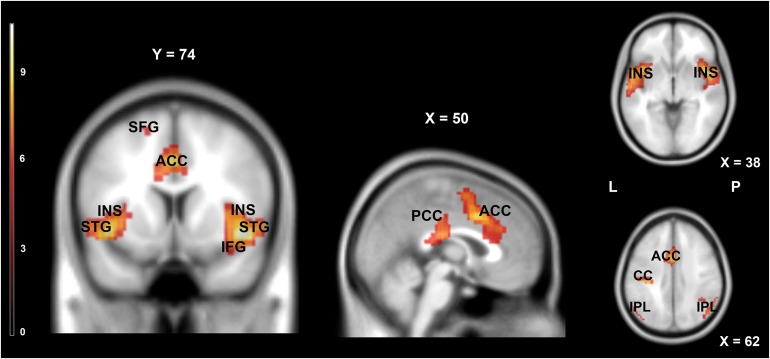
Brain activity pattern for the whole group (*n* = 61) associated with the main effect of rest (rest vs. movies). ACC, anterior cingulate cortex; CC, cingulate cortex; IFG, inferior frontal gyrus; INS, insula; IPL, inferior parietal lobule; PCC, posterior cingulate cortex; STG, superior temporal gyrus; SFG, superior frontal gyrus; L, left side; R, right side.

Figure 3 and Table 2 show the main effect of movie-watching (all categories of videos together vs. rest). As expected, this contrast revealed increased activations in visual, multisensory, and frontal areas as well as limbic structures. Significant bilateral activations were observed in the primary visual cortex, middle occipital gyrus, inferior occipital gyrus, superior occipital gyrus, cuneus, precuneus, lingual gyrus, fusiform gyrus, middle temporal gyrus, inferior temporal gyrus, posterior cingulate cortex, hippocampus, parahippocampal gyrus, thalamus, inferior parietal lobule, superior parietal lobule, and medial frontal gyrus. The opposite contrast (rest vs. all movies) showed widespread activations typically associated with default mode network (DMN). Detailed results are shown in Figure 4 and Table 3.

**TABLE 2:** Areas significantly more activated during movies watching (movies vs. rest).

			**Coordinates**				
**Structures**		**BA**	***x***	***y***	***z***	***t* (peak)**	**Cluster size (*k*)**
Middle occipital gyrus	R	18	25	−94	3	16.01	6322
Lingual gyrus			4	−85	−9	15.83	
			25	−94	−6	15.73	
Inferior frontal gyrus	R	6	43	5	27	7.58	194
			46	29	15	6.86	
			43	14	24	6.71	
Precuneus	L	7	−23	−58	57	9.24	150
Cingulate gyrus	L	31	−14	−25	36	8.50	36
Superior frontal gyrus	L	8	−23	32	51	6.96	32
			−14	38	51	6.49	
Medial frontal gyrus	R	6	22	−4	51	6.27	18
Medial frontal gyrus	L	32	−11	41	−15	5.53	12
Postcentral gyrus	R	1	25	−37	45	5.47	11
Precentral gyrus			31	−34	54	5.44	
Cingulate gyrus	R	31	13	−22	39	5.75	8

*MNI coordinates, Brodmann areas (BAs), peak activation, and volume are presented at the estimated significance threshold: t-values and cluster size (k).*

**TABLE 3:** Areas significantly more activated during rest (rest vs. movies).

			**Coordinates**				
**Structures**		**BA**	***x***	***y***	***z***	***t* (peak)**	**Cluster size (*k*)**
Insula	R	13	46	8	−9	11.61	666
Superior temporal gyrus			43	38	−15	7.62	
Inferior frontal gyrus			37	32	−12	7.36	
Superior temporal gyrus	L	13	−44	11	−9	9.64	555
Insula			−53	5	0	8.35	
Cingulate gyrus	L	32	−2	11	39	9.30	474
			−2	−1	57	7.23	
			1	32	27	6.47	
Postcentral gyrus	L	1	−35	−25	42	9.21	306
Inferior parietal lobule	R	39	46	−61	48	7.76	139
Posterior cingulate cortex	R	23	1	−22	21	7.39	96
Inferior frontal gyrus	L	47	−44	35	−18	7.03	61
Hippocampus	L	30	−17	−40	9	6.77	34
Inferior parietal lobule	L	39	−56	−58	39	6.10	16
Supramarginal gyrus	L	39	−38	−49	36	6.26	12
Putamen	R	49	19	5	−9	7.06	12

*MNI coordinates, Brodmann areas (BAs), peak activation, and volume are presented at the estimated significance threshold: t-values and cluster size (k).*

#### Effects of Movies Categories

To ensure that the manipulation of the movie content was effective, and systematically related not only to the behavioral ratings but also brain activation patterns, we run a series of contrasts comparing all pairs of video categories.

**FIGURE 5:**
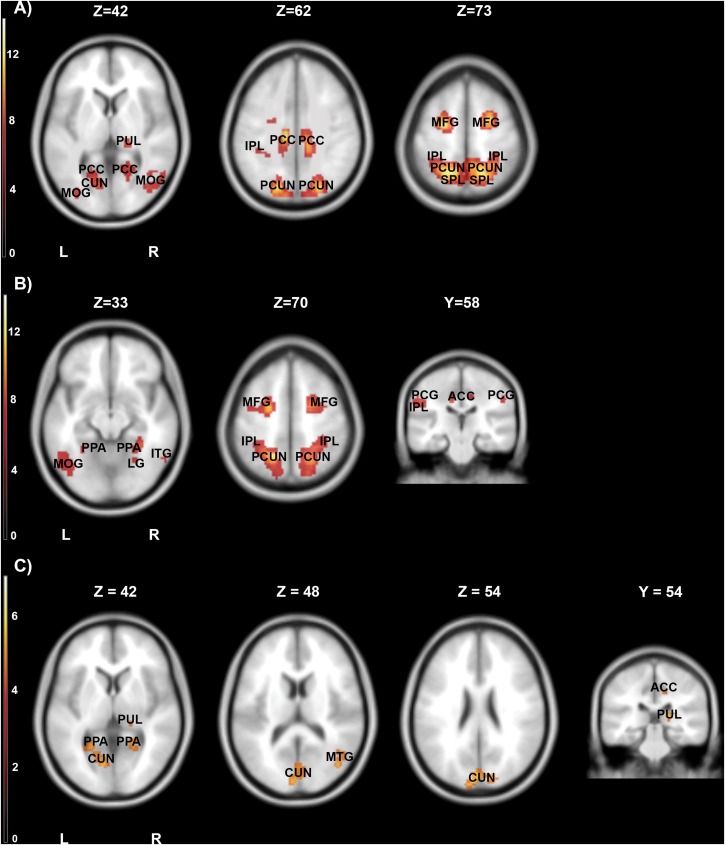
Brain activity pattern for the whole group (*n* = 61) associated with the effects of movies categories: **(A)** high arousing movies vs. neutral movies, **(B)** high arousing movies vs. low arousing movies, **(C)** low arousing movies vs. neutral movies. ACC, anterior cingulate cortex; CUN, cuneus; IPL, inferior parietal lobule; ITG, inferior temporal gyrus; LG, lingual gyrus; MFG, middle frontal gyrus; MOG, middle occipital gyrus; PCC, posterior cingulate cortex; PCG, postcentral gyrus; PCUN, precuneus; PPA, parahippocampal gyrus; PUL, pulvinar; SPL, superior parietal lobule; L, left side; R, right side.

The first contrast compared high arousing and neutral movies. It revealed widespread activations in areas responsible for the processing of emotional stimuli (inferior parietal lobule, cingulate cortex, middle frontal gyrus), visual perception (thalamus) and parahippocampal gyrus. Detailed results are showed in Table 4 and Figure 5A.

**TABLE 4:** Areas significantly more activated by high arousing movies in comparison with neutral movies (high arousing movies vs. neutral movies).

			**Coordinates**				
**Structures**		**BA**	***x***	***y***	***z***	***t* (peak)**	**Cluster size (*k*)**
Cingulate gyrus	L	31	−14	−25	36	12.57	3500
Precuneus	R	7	16	−55	60	11.43	
Middle frontal gyrus	R	6	25	2	57	10.34	334
Lingual gyrus	R	19	22	−61	−9	8.51	289
Fusiform gyrus			28	−49	−9	7.62	
Parahippocampal gyrus			19	−55	0	6.96	
Middle frontal gyrus	L	6	−23	−7	57	11.24	273
			−14	−7	63	8.45	
Middle temporal gyrus	L	37	−53	−67	−6	8.56	107
Lingual gyrus	L	17	−14	−70	3	7.23	91
Parahippocampal gyrus	L	37	−29	−52	−9	6.63	47
Inferior parietal lobule	L	40	−59	−28	33	6.77	41
Inferior frontal gyrus	R	6	46	5	27	6.24	30
Thalamus	R	50	19	−28	3	7.31	18

*MNI coordinates, Brodmann areas (BAs), peak activation, and volume are presented at the estimated significance threshold: t-values and cluster size (k).*

In the second step, we contrasted high and low arousing movies. Results showed that viewing material depicting extreme sports was related to stronger activations in inferior parietal lobule and anterior cingulate cortex (Table 5A and Figure 5B).

**TABLE 5A:** Areas significantly more activated by high arousing movies in comparison with low arousing movies (high arousing movies vs. low arousing movies).

			**Coordinates**				
**Structures**		**BA**	***x***	***y***	***z***	***t* (peak)**	**Cluster size (*k*)**
Precuneus	L	7	−20	−58	54	12.95	1249
			−20	−79	42	8.84	
			−20	−82	30	8.68	
Superior parietal lobule	R	7	19	−52	63	10.81	1000
Precuneus			19	−64	54	9.17	
			40	−82	15	8.50	
Middle frontal gyrus	L	6	−23	−7	54	9.99	215
			−41	−7	51	6.07	
Middle frontal gyrus	R	6	22	−4	57	10.02	187
Cingulate gyrus	L	31	−11	−22	39	8.24	41
Middle temporal gyrus	R		46	−58	−6	7.28	
Parahippocampal gyrus	R	37	31	−43	−12	7.40	34
Inferior parietal lobule	L	40	−53	−25	33	7.05	57
Postcentral gyrus	R	40	55	−22	36	6.59	23
Parahippocampal gyrus	L	37	−29	−46	−15	6.49	18
Cingulate gyrus	R	31	13	−25	39	6.15	10
Precentral gyrus	L	6	−50	2	27	5.91	8
Fusiform gyrus	R	37	28	−61	−9	5.74	8
Inferior frontal gyrus	R	44	49	5	24	5.52	6

Not surprisingly, the activations evoked by low arousing (vs. neutral) movies were weaker than those observed in the first contrast. Significant activity was present in visual processing structures (precuneus, cuneus and thalamus), middle frontal, and parahippocampal gyri (Table 5B and Figure 5C).

**TABLE 5B:** Areas significantly more activated by low arousing movies in comparison with neutral movies (low arousing movies vs. neutral movies).

			**Coordinates**				
**Structures**		**BA**	***x***	***y***	***z***	***t* (peak)**	**Cluster size (*k*)**
Precuneus	R	31	13	−46	51	8.70	305
			7	−49	57	8.04	
Cuneus	L	18	−5	−91	18	7.35	225
			−8	−94	27	6.71	
Lingual gyrus	L	18	−11	−70	3	6.72	108
			−23	−52	0	6.37	
Middle temporal gyrus	R	19	43	−73	15	6.37	44
Parahippocampal gyrus	R	18	22	−46	0	6.93	42
Thalamus	R	50	16	−31	6	6.86	20
Cingulate gyrus	L	23	−11	−22	36	6.37	17
Middle frontal gyrus	L	6	−17	−7	63	6.10	12
Middle frontal gyrus	R	6	16	−10	66	6.14	5

*MNI coordinates, Brodmann areas (BAs), peak activation, and volume are presented at the estimated significance threshold: t-values and cluster size (k).*

#### Temperament – BOLD Relationship

The relationship between the temperamental traits and brain activity while watching low and high arousing movies was explored using ROI analysis. It revealed a markedly different pattern of correlations depending on the stimulus category.

For high arousing movies, we observed negative relationships of β values in the middle orbitofrontal gyrus with perseverance and emotional reactivity scale. A positive correlation was revealed for activity scale in the same brain area. Furthermore, the BOLD signal in the insula and hippocampus negative correlated with scores on endurance scale. Detailed results are presented in Table 6.

**TABLE 6:** Pearson correlation coefficients between ß values of BOLD signal in ROIs and raw temperamental scores of perseverance, emotional reactivity, endurance, and activity scales in high and low arousing movies condition.

**High-arousing movies**		**Temperamental traits**			
**Structure**		**Perseverance**	**Emotional reactivity**	**Endurance**	**Activity**
Medial orbitofrontal gyrus	L	−0.272^*^	−0.321^*^	−0.10	0.267^*^
	R	−0.072	−0.101	−0.058	0.040
Hippocampus	L	0.060	0.035	−0.195	0.114
	R	−0.083	−0.015	−0.317^*^	0.160
Insula	L	−0.018	−0.166	−0.237	0.061
	R	−0.112	−0.125	−0.300^*^	0.082
Lingual gyrus	L	−0.060	−0.060	−0.367^∗∗^	−0.050
	R	−0.086	0.008	−0.394^∗∗^	−0.038

**Low-arousing movies**		**Temperamental traits**			
**Structure**		**Perseverance**	**Emotional reactivity**	**Endurance**	**Activity**

Amygdala	L	−0.005	0.356^∗∗^	−0.107	−0.051
	R	−0.058	0.320^*^	−0.092	0.059
Caudate	L	0.172	0.259	−0.001	0.101
	R	0.135	0.273^*^	−0.003	0.071
Inferior orbitofrontal gyrus	L	−0.062	0.200	0.103	−0.026
	R	−0.027	0.271^*^	0.052	−0.044
Inferior frontal gyrus	L	−0.012	0.183	0.060	−0.098
	R	−0.044	0.323^*^	0.128	−0.117
Medial frontal gyrus	L	0.027	0.136	0.039	−0.032
	R	0.030	0.271^*^	0.073	−0.146
Superior frontal gyrus	L	0.016	0.062	0.093	−0.006
	R	0.018	0.296^*^	0.191	−0.162
Hippocampus	L	0.120	0.360^∗∗^	−0.073	−0.039
	R	−0.037	0.152	−0.192	0.154
Precuneus	L	−0.023	0.231	−0.090	−0.026
	R	−0.023	0.299^*^	−0.081	−0.074

^*^p < 0.05, ^∗∗^p < 0.01.

**FIGURE 6:**
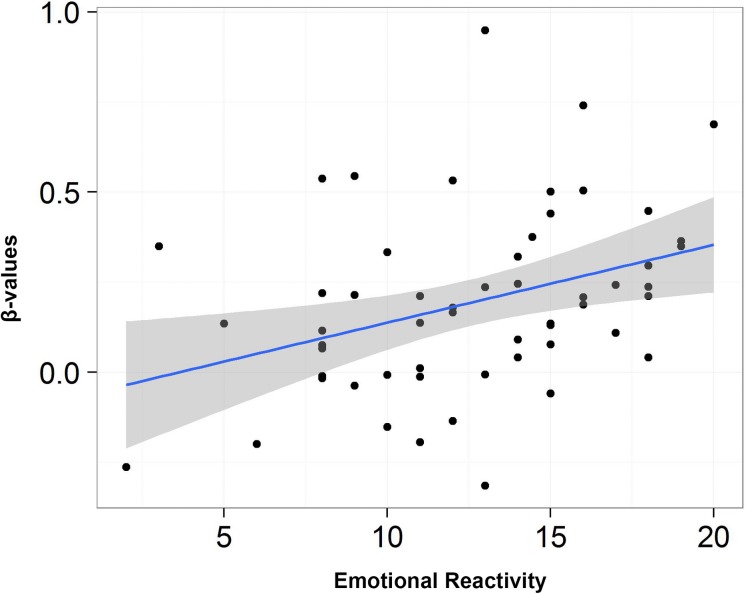
Scatterplot depicting the correlation between scores from emotional reactivity and β-values for low arousing movies condition in left amygdala.

A similar analysis for low arousing movies revealed very interesting results for the emotional reactivity scale which correlated with the key structures of the limbic system: amygdala (Figure 6), hippocampus, and inferior orbitofrontal gyrus, as well as caudate and precuneus and frontal cortex. Detailed results are presented in Table 6.

## Discussion

Before referring to the obtained results, we would like to underline two important novel aspects of our study. Firstly, it employed a new procedure aiming to reveal the neural correlates of emotional stimuli processing while controlling the stimulatory value of the videos presented during the fMRI session. That allowed us to identify brain-activity correlates of temperamental traits across varying arousal levels. As mentioned before, RTT states explicitly that temperament plays a regulative role in providing levels of stimulation suiting our individual needs (Strelau, 2009). Together with the well-established connection between facets of temperament defined by RTT and engagement in activities varying in stimulatory levels (including extreme sports, see: Boldak and Guszkowska, 2012), it provided a strong theoretical background justifying our choice of stimuli. Secondly, to the best of our knowledge, our study is the first ever attempt to investigate the complex interplay between temperamental traits conceptualized by RTT, levels of arousal and brain activity.

### The Validity of the Stimuli

We used an experimental design in which movies evoking varying levels of arousal were chosen in order to show the moderating role of temperament on brain activity. Our behavioral data confirmed that selected movie material differed significantly on the scales of basic emotions, valence, and arousal. As expected, the most arousing movies got the highest scores on arousal and fear scales. Surprisingly, this category of stimuli also scored highest on the happiness scale. Buckley (2016) suggests that this mixture of emotions while watching or engaging in extreme sports is quite typical. Thrill and exhilaration are often reported by adventure practitioners (e.g., Cater, 2006; Tsaur et al., 2013), and they are coupled with the successful performance of demanding activities under risk (Buckley, 2012).

The validity of stimulus selection was further confirmed by the results of the fMRI analysis. As expected, significant differences in brain activity were observed depending on the stimulative properties of the movies. All conducted contrasts comparing movie categories revealed expected results. The main effect of movies and rest showed activation patterns that were almost identical to those reported by Eryilmaz et al. (2011). Highly arousing movies evoked widespread activations in sensory and limbic brain regions, reflecting attentional engagement and expected an emotional response. Increased activation in limbic, subcortical, motor cortex and top-down attention areas related to higher arousal levels might be convincingly explained by the complex, and dynamic features of stimuli, as well as the strength of affective response, revealed in behavioral assessment of the movies. Importantly, our results are consistent with the studies where procedures with dynamic stimuli (diverse in terms of the arousal levels and specific emotional categories) and extreme sports were used (e.g., Hasson et al., 2010; Keysers et al., 2010; Nummenmaa et al., 2012; Goldberg et al., 2014; Huis in ’t Veld and De Gelder, 2015; Morawetz et al., 2015).

### Temperamental Traits

According to RTT, extreme sports are supposed to be appetitive stimuli for subjects with low emotional reactivity and high activity scores. Thus, we expected a positive correlation between activity scale and activations of structures in the reward system: nucleus accumbens, amygdala and orbitofrontal cortex. On the other hand, we predicted the same category of stimuli to be aversive for high emotionally reactive subjects. This prediction was based on the findings indicating that in subjects watching aversive stimuli neuroticism is associated with increased activation in brain regions involved in general emotion processing, including the middle cingulate gyrus and dorsal (medial) prefrontal cortex (Servaas et al., 2013). As emotional reactivity is highly correlated with neuroticism (Zawadzki and Strelau, 1997), we expected a similar pattern of correlations in highly arousing conditions in our study.

The obtained results did not provide support for the hypotheses involving activity trait. The only positive correlation in high arousing sports condition was observed in medial orbitofrontal gyrus. Interestingly, negative correlation with the BOLD signal in the same brain area was observed for perseverance and emotional reactivity. This result is particularly interesting because of the postulated role of the orbitofrontal cortex. Cavada and Schultz (2000) suggested that this structure is crucial in affect regulation and social adaptation – main processes defining both temperamental and personality traits. Winecoff et al. (2013) compared activation of the ventromedial prefrontal cortex (vmPFC) in response to viewing affective pictures and confirmed that it is associated with the processing of positive emotions. Furthermore, it is known from animal studies, that orbitofrontal cortex is engaged in reward processing and discrimination. Iversen and Mishkin (1970) showed that monkeys with lesions of the orbitofrontal cortex were impaired relative to controls in retaining an auditory frequency differentiation and in learning object and spatial reversals and also exhibited symptoms of affect deregulation. In a more recent study, researchers showed that human brain lesions data also support the central role of vmPFC in reward expectation and evaluation (Manohar and Husain, 2016).

In the context of the present study based on video stimuli, it is important to underline the connection between orbitofrontal cortex (OFC) with the thalamus and visual cortex. Researchers showed that OFC plays an important role in positive evaluation of visual stimuli (Thorpe et al., 1983; Rolls, 1996). At the same time, OFC activation has been linked to openness to experience (Wenfu et al., 2014), a trait which correlates positively with activity measured by FCB-TI questionnaire (Zawadzki and Strelau, 1997). To sum up, we hypothesize that OFC activity during the presentation of arousing movies plays an important role in the positive evaluation of perceived stimuli in subjects with higher scores on activity scale. At the same time, we postulate the reverse to be true for emotional reactivity, increased levels of this trait predict lower OFC activations.

Other results for high arousing movies showed a negative correlation between BOLD signal and endurance in the hippocampus, insular cortex, and lingual gyrus. This result is coherent with the theoretical characteristics of all these traits. RTT defines endurance as the ability to react adequately in situations demanding long-lasting or highly stimulating activity (Zawadzki and Strelau, 1997). The evaluative role of insula is well established (Berntson et al., 2011) and it is known to be involved in the contextualization of both positive and negative stimuli (Pollatos et al., 2007; Knutson and Greer, 2008; Rolls et al., 2009; Smith et al., 2009). Its activation has been reported in anticipation of both gains and losses, whereas lesions in insula disrupt performance in risky conditions (Knutson and Greer, 2008). On the other hand, the hippocampus plays a crucial role in learning about the emotional significance of the presented stimuli (Phelps, 2004). In light of the abovementioned results, our study strongly suggests that high-endurance is related to lower levels of activity in the structures responsible for processing of emotional stimuli, allowing to be more resilient in situations characterized by intense or long-lasting stimulation.

Emotional reactivity trait scores correlated positively with the BOLD signal in the majority of the limbic system structures (including the amygdala) when participants were shown videos depicting sports activities with low stimulative value. These results fully align with predictions of major temperamental theories. Emotional reactivity scale correlates highly and positively with neuroticism as it is defined by both PEN (Eysenck and Eysenck, 1985) and Big Five (McCrae and Costa, 1987). Well before the era of modern human brain imaging, Hans Eysenck proposed a theory (Eysenck, 1967) that neuroticism is explained by differences in the level of activity primarily in the limbic system. Researchers were able to show stronger activations connected to high scores in neuroticism scales while watching negative stimuli in: middle frontal gyrus (upsetting scenes, Canli et al., 2001), medial PFC (sad faces, Haas et al., 2008), anterior cingulate (“negative” upsetting scenes, Haas et al., 2007), temporal pole (sad faces, Jimura et al., 2009), amygdala (“negative” upsetting scenes, Cunningham et al., 2010), and the basal ganglia (sad emotion symbols, Bruhl et al., 2011). In the current study, we corroborate these finding showing that scenes depicting low-arousing sports offer an opportunity to differentiate groups defined by reactivity scores based on their levels of BOLD signal in limbic system structures.

### Limitations of the Study

It is important to underline that some of the presented conclusions require corroboration in further research, due to the limitations of our study. Most importantly, our findings are based on data obtained in the female-only sample. As explained previously, our decision not to include male participants was motivated by the need to maximize the variance of key temperamental characteristics while controlling for the potential confounding effects of sex. At the same time, this approach allowed as to use raw scores obtained from FCB-TI in all correlational analysis, without relying on test norms.

The second important limitation of our approach stems from the moderate size of our study sample. Additionally, both estimates of fMRI activations and questionnaire measurements offer limited reliability, which negatively affects theoretical upper limits of correlations between activity scores and temperamental traits. These premises motivated our decision to report uncorrected *p*-values in all correlational analyses. We are fully aware of the fact that it is possible that some of the reported significant results might be false-positives, however, given limited power and coherent pattern of key results (effects of reactiveness in low-arousal condition and activity for high-arousal movies), we find this approach justified.

## Conclusion

The experimental procedure employed in our study allows to investigate the brain correlates of highly ecologically valid video-stimuli eliciting varying levels of arousal. Firstly, following our expectations, we showed that brain activation patterns were systematically related to the stimulatory value of the presented material, with more pronounced reactions evoked by videos depicting extreme-sports activities. Secondly, correlational analysis revealed an interesting pattern of relationships between temperamental traits and BOLD signal levels. Our results showed that orbitofrontal cortex responds differently to high arousing movie stimuli depending on temperament of the subject. Additionally, we identified a link between high emotional reactivity and activation of the limbic structures providing first ever validation of RTT theory based on fMRI data.

It is also worth noting that our results bear some important methodological implications and indicate that controlling for temperamental differences is crucial in research aiming at identification of brain correlates of emotional processes. Whereas most of the published studies focus on controlling the affective aspects of the stimuli, our research shows that individual differences and stimulative properties might play a significant moderating role and determine the evaluative interpretations construed by the subjects. For example, for individuals with high activity scores, extreme sports are appetitive, whereas for reactive subjects the same material might induce anxiety. As suggested by the title of this article, manipulation of perceived risk and stress levels and using close-to-real-life video stimuli might offer a new (and exciting!) avenue for researchers interested in the study of individual differences and brain underpinnings of emotional reactions.

## Ethics Statement

This study was carried out in accordance with the recommendations of a local research ethics committee at the SWPS University of Social Sciences and Humanities with written informed consent from all subjects. All subjects gave written informed consent in accordance with the Declaration of Helsinki. The protocol was approved by the ethics committee at the SWPS University of Social Sciences and Humanities.

## Author Contributions

MarB, MakB, JS, and MK made substantial contributions to the conception or design of the study. MarB, PS, and AK acquired the data. MarB and MakB analyzed the data. MarB, MakB, and MK interpreted the data. MarB drafted the manuscript. MakB, PS, AK, JS, and MK critically revised the manuscript for important intellectual content. All authors approved the final version of the manuscript and agreed to be accountable for all aspects of the work in ensuring that questions related to the accuracy or integrity of any part of the work are appropriately investigated and resolved.

## Conflict of Interest Statement

The authors declare that the research was conducted in the absence of any commercial or financial relationships that could be construed as a potential conflict of interest.

## Footnotes

1 http://exp.lobi.nencki.gov.pl

2 https://www.jamovi.org

3 http://www.nordicneurolab.com/

4 http://www.gin.cnrs.fr/en/tools/aal-aal2/

## References

[B1] Adelstein J. S. Shehzad Z. Mennes M. DeYoung C. G. Zuo X. N. Kelly C. (2011). Personality is reflected in the brain’s intrinsic functional architecture. *PLoS One* 6:e27633. 10.1371/journal.pone.0027633 22140453 PMC3227579

[B2] Berntson G. G. Norman G. J. Bechara A. Bruss J. Tranel D. Cacioppo J. T. (2011). The insula and evaluative processes. *Psychol. Sci.* 22 80–86. 10.1177/0956797610391097 21148459 PMC3261800

[B3] Boldak A. Guszkowska M. (2012). Are Skydivers a homogenous group? Analysis of features of temperament, sensation seeking, and risk taking. *Int. J. Aviat. Psychol.* 23 197–212. 10.1080/10508414.2013.799342 23795909

[B4] Brett M. Anton J. L. Valabregue R. Poline J. B. (2002). “Region of interest analysis using an SPM toolbox [abstract],” in *Proceedings of the 8th International Conference on Functional Mapping of the Human Brain*, Sendai.

[B5] Bruhl A. B. Viebke M. C. Baumgartner T. Kaffenberger T. Herwig U. (2011). Neural correlates of personality dimensions and affective measures during the anticipation of emotional stimuli. *Brain Imaging Behav.* 5 86–96. 10.1007/s11682-011-9114-7 21264550

[B6] Buckley R. C. (2012). Rush as a key motivation in skilled adventure tourism: resolving the risk recreation paradox. *Tour Manag.* 33 961–970. 10.1016/j.tourman.2011.10.002

[B7] Buckley R. C. (2016). Qualitative analysis of emotions: fear and thrill. *Front. Psychol.* 7:1187. 10.3389/fpsyg.2016.01187 27559323 PMC4978710

[B8] Buss A. H. Plomin R. (1975). *A Temperament Theory of Personality Development.* New York, NY: Wiley-Interscience.

[B9] Buss A. H. Plomin R. (1984). *Temperament: Early Developing Personality Traits.* London: Psychology Press.

[B10] Canli T. Sivers H. Whitfield S. L. Gotlib I. H. Gabrieli J. D. (2002). Amygdala response to happy faces as a function of extraversion. *Science* 296:2191 10.1126/science.1068749 12077407

[B11] Canli T. Zhao Z. Desmond J. E. Kang E. Gross J. Gabrieli J. D. (2001). An fMRI study of personality influences on brain reactivity to emotional stimuli. *Behav. Neurosci.* 115 33–42. 10.1037//0735-7044.115.1.33 11256451

[B12] Cater C. I. (2006). Playing with risk? Participant perceptions of risk and management implications in adventure tourism. *Tour Manag.* 27 317–325. 10.1016/j.tourman.2004.10.005

[B13] Cavada C. Schultz W. (2000). The Mysterious orbitofrontal cortex. *Cereb. Cortex* 10:205 10.1093/cercor/10.3.205 10731216

[B14] Costa P. T. McCrae R. R. (1980). Influence of extraversion and neuroticism on subjective well- being: happy and unhappy people. *J. Pers. Soc. Psychol.* 38 668–678. 10.1037//0022-3514.38.4.668 7381680

[B15] Cremers H. R. Demenescu L. R. Aleman A. Renken R. Van Tol M. J. Van Der Wee N. J. (2009). Neuroticism modulates amygdala-prefrontal connectivity in response to negative emotional facial expressions. *Neuroimage* 49 963–970. 10.1016/j.neuroimage.2009.08.023 19683585

[B16] Cunningham W. A. Arbuckle N. L. Jahn A. Mowrer S. A. Abduljalil A. M. (2010). Aspects of neuroticism and the amygdala: chronic tuning from motivational styles. *Neuropsychologia* 48 3399–3404. 10.1016/j.neuropsychologia.2010.06.026 20600183

[B17] Dragan W. Ł Oniszczenko W. (2005). Polymorphisms in the serotonin transporter gene and their relationship to two temperamental traits measured by the formal characteristics of behavior- Temperament inventory: activity and emotional reactivity. *Neuropsychobiology* 51 269–274. 10.1159/000085823 15905633

[B18] Dragan W. Ł Oniszczenko W. (2006). Associaction of a functional polymporphism in the serotonin transporter gene with personality traits in females in Polish population. *Neuropsychobiology* 54 45–50. 10.1159/000095741 16966839

[B19] Dreher J. C. Kohn P. Berman K. F. (2006). Neural coding of distinct statistical properties of reward information in humans. *Cereb. Cortex* 16 561–573. 10.1093/cercor/bhj004 16033924

[B20] Eliasz A. (1981). *Temperament a System Regulacji Stymulacji [Temperament and Stimulation Regulation System].* Warszawa: PWN.

[B21] Eryilmaz H. Van De Ville D. Schwartz S. Vuilleumier P. (2011). Impact of transient emotions on functional connectivity during subsequent resting state: a wavelet correlation approach. *Neuroimage* 54 2481–2491. 10.1016/j.neuroimage.2010.10.021 20955802

[B22] Eysenck H. J. (1947). *Dimensions of Personality.* London: Routledge & Kegan Paul.

[B23] Eysenck H. J. (1967). *The Biological Basis of Personality*. Springfield, IL: Thomas, 100–117.

[B24] Eysenck H. J. (1973). *The Measurement of Intelligence.* Baltimore, Md: Williams & Wilkins.

[B25] Eysenck H. J. Eysenck M. W. (1985). *Personality and Individual Differences: A Natural Science Approach.* New York, NY: Plenum Press.

[B26] Frank T. C. Kim G. L. Krzemien A. Van Vugt D. A. (2010). Effect of menstrual cycle phase on corticolimbic brain avtivation by visual food cues. *Brain Res.* 1363 81–92. 10.1016/j.brainres.2010.09.071 20920491

[B27] Gingnell M. Morell A. Bannbers E. Wikström J. Sundström Poromaa I. (2012). Menstrual cycle effects on amygdala reactivity to emotional stimulation in premenstrual dysphoric disorder. *Horm. Behav.* 62 400–406. 10.1016/j.yhbeh.2012.07.005 22814368

[B28] Glenc M. (2006). “Skłonnośæ do podejmowania ryzyka czyli psychologiczna charakterystyka ryzykantów,” [Risk taking proneness—The psychological portrait of risk takers),” in *Psychologia zachowań ryzykownych— koncepcje, badania, praktyka*, eds Goszczyńska M. Studenski R. (Warszawa: Wydawnictwo Akademickie Żak), 216–235.

[B29] Goldberg H. Preminger S. Malach R. (2014). The emotion-action link? Naturalistic emotional stimuli preferentially activate the human dorsal visual stream. *Neuroimage* 84 254–264. 10.1016/j.neuroimage.2013.08.032 23994457

[B30] Goldsmith H. H. Buss A. H. Plomin R. Rothbart M. K. Thomas A. Chess S. (1987). Roundtable: what is temperament? Four approaches. *Child Dev.* 58 505–529. 10.1111/j.1467-8624.1987.tb01398.x 3829791

[B31] Goldsmith H. H. Campos J. J. (1982). “Toward a theory of infant temperament,” in *The Development of Attachment and Affiliative Systems*, eds Emde A. N. Harmon R. J. (New York, NY: Plenum), 161–193. 10.1007/978-1-4684-4076-8_13

[B32] Gracz J. Sankowski T. (2000). *Psychologia sportu [Sport psychology].* Poznan: Akademia Wychowania Fizycznego.

[B33] Gross J. J. Levenson R. W. (1995). Emotion elicitation using films. *Cogn. Emot.* 9 87–108. 10.1080/02699939508408966

[B34] Haas B. W. Constable R. T. Canli T. (2008). Stop the sadness: neuroticism is associated with sustained medial prefrontal cortex response to emotional facial expressions. *Neuroimage* 42 385–392. 10.1016/j.neuroimage.2008.04.027 18511299 PMC2789588

[B35] Haas B. W. Omura K. Constable R. T. Canli T. (2007). Emotional conflict and neuroticism: personality-dependent activation in the amygdala and subgenual anterior cingulate. *Behav. Neurosci.* 121 249–256. 10.1037/0735-7044.121.2.249 17469914

[B36] Hasson U. Malach R. Heeger D. J. (2010). Reliability of cortical activity during natural stimulation. *Trends Cogn. Sci.* 14 40–48. 10.1016/j.tics.2009.10.011 20004608 PMC2818432

[B37] Huis in ’t Veld E. M. J. De Gelder B. (2015). From personal fear to mass panic: the neurological basis of crowd perception. *Hum. Brain Mapp.* 36 2338–2351. 10.1002/hbm.22774 25716010 PMC6869145

[B38] Iversen S. D. Mishkin M. (1970). Perseverative interference in monkeys following selective lesions of the inferior prefrontal convexity. *Exp. Brain Res.* 11 376–386. 4993199 10.1007/BF00237911

[B39] Jimura K. Konishi S. Miyashita Y. (2009). Temporal pole activity during perception of sad faces, but not happy faces, correlates with neuroticism trait. *Neurosci. Lett.* 453 45–48. 10.1016/j.neulet.2009.02.012 19429013

[B40] Kagan J. Reznick J. S. Snidman N. (1988). Biological bases of childhood shyness. *Science* 240 167–171. 10.1126/science.3353713 3353713

[B41] Kehoe E. G. Toomey J. M. Balsters J. H. Bokde A. L. W. (2011). Personality modulates the effects of emotional arousal and valence on brain activation. *Soc. Cogn. Affect Neurosci.* 7 858–870. 10.1093/scan/nsr059 21948954 PMC3475359

[B42] Kennis M. Rademaker A. R. Geuze E. (2013). Neural correlates of personality: an integrative review. *Neurosci. Biobehav. Rev.* 37 73–95. 10.1016/j.neubiorev.2012.10.012 23142157

[B43] Keysers C. Kaas J. H. Gazzola V. (2010). Somatosensation in social perception. *Nat. Rev. Neurosci.* 11 417–428. 10.1038/nrn2833 20445542

[B44] Klonowicz T. (1984). *Reaktywność a funkcjonowanie człowieka w różnych warunkach stymulacyjnych [Reactivity and Functionning in Different Conditions of Stimulation].* Warszawa: Zakład Narodowy Ossolińskich.

[B45] Knutson B. Greer S. M. (2008). Anticipatory affect: neural correlates and consequences for choice. *Philos. Trans. R. Soc. Lond. B Biol. Sci.* 363 3771–3786. 10.1098/rstb.2008.0155 18829428 PMC2607363

[B46] Manohar S. G. Husain M. (2016). Human ventromedial prefrontal lesions alter incentivisation by reward. *Cortex* 76 104–120. 10.1016/j.cortex.2016.01.005 26874940 PMC4786053

[B47] McCrae R. R. Costa P. T. (1987). Validation of the five factor model of personality across instruments and observers. *J. Pers. Soc. Psychol.* 52 81–90. 10.1037/0022-3514.52.1.81 3820081

[B48] Miller E. K. Cohen J. D. (2001). An integrative theory of prefrontal cortex function. *Annu. Rev. Neurosci.* 24 167–202. 10.1146/annurev.neuro.24.1.167 11283309

[B49] Morawetz C. Bode S. Baudewig J. Kirilina E. Heekeren H. R. (2015). Changes in effective connectivity between dorsal and ventral prefrontal regions moderate emotion regulation. *Cereb. Cortex* 26 1923–1937. 10.1093/cercor/bhv005 25631055

[B50] Nummenmaa L. Glerean E. Viinikainen M. Jaaskelainen I. P. Hari R. Sams M. (2012). Emotions promote social interaction by synchronizing brain activity across individuals. *Proc. Natl. Acad. Sci. U.S.A.* 109 9599–9604. 10.1073/pnas.1206095109 22623534 PMC3386135

[B51] Oniszczenko W. Dragan W. Ł (2012). Association between temperament in terms of the Regulative Theory of Temperament and DRD4 and DAT1 gene polymorphisms. *Compr. Psychiatry* 53 789–796. 10.1016/j.comppsych.2012.01.001 22342155

[B52] Paulus M. P. Rogalsky C. Simmons A. Feinstein J. S. Stein M. B. (2003). Increased activation in the right insula during risk-taking decision making is related to harm avoidance and neuroticism. *Neuroimage* 19 1439–1448. 10.1016/s1053-8119(03)00251-9 12948701

[B53] Phelps E. A. (2004). Human emotion and memory: interactions of the amygdala and hippocampal complex. *Curr. Opin. Neurobiol.* 14 198–202. 10.1016/j.conb.2004.03.015 15082325

[B54] Pollatos O. Gramann K. Schandry R. (2007). Neural systems connecting interoceptive awareness and feelings. *Hum. Brain Mapp.* 28 9–18. 10.1002/hbm.20258 16729289 PMC6871500

[B55] Rolls E. T. (1996). The orbitofrontal cortex. *Philos. Trans. R. Soc. Lond. B Biol. Sci.* 351 1433–1443. 10.1098/rstb.1996.0128 8941955

[B56] Rolls E. T. Grabenhorst F. Parris B. A. (2009). Neural systems underlying decisions about affective odors. *J. Cogn. Neurosci.* 22 1069–1082. 10.1162/jocn.2009.21231 19320548

[B57] Rothbart M. K. Derryberry D. (1981). “Development of individual differences in temperament,” in *Advances in Developmental Psychology*, Vol. 1, eds Lamb M. E. Brown A. (Hillsdale, NJ: Lawrence Erlbaum Associates), 37–86.

[B58] Rottenberg J. Ray R. R. Gross J. J. (2007). “Emotion elicitation using films,” in *The Handbook of Emotion Elicitation and Assessment*, eds Coan J. A. Allen J. J. B. (New York, NY: Oxford University Press), 9–28.

[B59] Sato W. Yoshikawa S. (2007). Enhanced experience of emotional arousal in response to dynamic facial expressions. *J. Nonverbal Behav.* 31 119–135. 10.1007/s10919-007-0025-7

[B60] Schaefer A. Nils F. F. Sanchez X. Philippot P. (2010). Assessing the effectiveness of a large database of emotion-eliciting films: a new tool for emotion researchers. *Cogn. Emot.* 24 1153–1172. 10.1080/02699930903274322

[B61] Servaas M. N. van der Velde J. Costafreda S. G. Horton P. Ormel J. Riese H. (2013). Neuroticism and the brain: a quantitative meta-analysis of neuroimaging studies investigating emotion processing. *Neurosci. Biobehav. Rev.* 37 1518–1529. 10.1016/j.neubiorev.2013.05.005 23685122

[B62] Shimamura A. P. Marian D. E. Haskins A. L. (2013). Neural correlates of emotional regulation while viewing films. *Brain Imaging Behav.* 7 77–84. 10.1007/s11682-012-9195-y 22843102

[B63] Smith B. W. Mitchell D. G. Hardin M. G. Jazbec S. Fridberg D. Blair R. J. (2009). Neural substrates of reward magnitude, probability, and risk during a wheel of fortune decision- making task. *Neuroimage* 44 600–609. 10.1016/j.neuroimage.2008.08.016 18804540 PMC2695942

[B64] Strelau J. (1982). “Kwestionariusz Temperamentu w konwencji typologicznej według Iwana P. Pawłowa,” in *Regulacyjne Funkcje Temperamentu*, ed. Strelau J. (Wrocław: Ossolineum), 205–236.

[B65] Strelau J. (1985). *Temperament – osobowośæ – działanie.* Warszawa: PWN.

[B66] Strelau J. (1998). *Temperament: A psychological perspective.* New York, NY: Plenum Press.

[B67] Strelau J. (2009). *Psychologia temperamentu [Psychology of temperament].* Warszawa: PWN.

[B68] Strelau J. Zawadzki B. (1993). The Formal Characteristics of Behaviour-Temperament Inventory (FCB-TI): theoretical assumptions and scale construction. *Eur. J. Pers.* 7 313–336. 10.1002/per.2410070504

[B69] Strelau J. Zawadzki B. Oniszczenko W. Angleitner A. Riemann R. (2002). Genetic and environmental determinants of emotions: data based on cross-country twin studies on temperament. *Polish Psychol. Bull.* 33 5–13.

[B70] Studenski R. (2004). *Ryzyko i ryzykowanie [Risk and risk taking].* Katowice: Wydawnictwo Uniwersytetu Śąskiego.

[B71] Terelak J. F. Jońca M. (2008). Aktywnośæ jako cecha temperamentu a strategie radzenia sobie ze stresem u pilotów wojskowych [Activity as temperament trait and coping strategies among army pilots]. *Polski Przegląd Medycyny Lotniczej.* 4 361–369.

[B72] Thomas A. Chess S. (1977). *Temperament and Development.* New York, NY: Brunner/Mazel.

[B73] Thorpe S. J. Rolls E. T. Maddison S. (1983). The orbitofrontal cortex: neuronal activity in the behaving monkey. *Exp. Brain Res.* 49 93–115. 6861938 10.1007/BF00235545

[B74] Tsaur S. H. Lin W. R. Liu J. S. (2013). Sources of challenge for adventure tourists: scale development and validation. *Tour Manage.* 38 85–93. 10.1016/j.tourman.2013.03.004

[B75] Wenfu L. Xueting L. Liljie H. Xiangzhen K. Wenjing Y. Dongtao W. (2014). Brain structure links trait creativity to openness to experience. *Soc. Cogn. Affect Neurosci.* 10 191–198. 10.1093/scan/nsu041 24603022 PMC4321617

[B76] Westermann R. Spies K. Stahl G. Hesse F. W. (1996). Relative effectiveness and validity ofmood induction procedures: a meta-analysis. *Eur. J. Soc. Psychol.* 26 557–580.

[B77] Windle M. Lerner R. M. (1986). Reassessing the dimensions of temperamental individuality across the life span:the revised dimensions of temperament survey (DOTS- R). *J. Adolesc. Res.* 1 213–229. 10.1177/074355488612007

[B78] Winecoff A. Clithero J. A. Carter R. M. Bergman S. R. Wang L. Huettel S. A. (2013). Ventromedial prefrontal cortex encodes emotional value. *J. Neurosci.* 33 11032–11039. 10.1523/JNEUROSCI.4317-12.2013 23825408 PMC3718369

[B79] Yarkoni T. (2015). “Neurobiological substrates of personality: A critical overview,”,” in *APA Handbooks in Psychology. APA Handbook of Personality and Social Psychology, Personality Processes and Individual Differences*, Vol. 4 eds Mikulincer M. Shaver P. R. Cooper M. L. Larsen R. J. (Washington, DC: American Psychological Association), 61–83.

[B80] Zawadzki B. Strelau J. (1995). Podstawy teoretyczne, konstrukcja i własności psychometryczne inwentarza: “Formalna Charakterystyka Zachowania – Kwestionariusz Temperamentu”. *Studia Psychologiczne* 33 49–95.

[B81] Zawadzki B. Strelau J. (1997). *Formalna Charakterystyka Zachowania-Kwestionariusz Temperamentu (FCZ-KT). Podrȩcznik. Warszawa: Pracownia Testów Psychologicznych PTP*.

